# Impact of COVID-19 on celebration of death, mortuary, and funerary customs in Bangladesh: A qualitative study

**DOI:** 10.1016/j.heliyon.2024.e30369

**Published:** 2024-04-27

**Authors:** Md Abdur Rahman, Md Redwanul Islam, Monidipa Chowdhury, Md. Asaduzzaman, Proloy Barua, M Atiqul Haque

**Affiliations:** a Department of Anthropology, Comilla University, Cumilla, 3500, Bangladesh; b Department of Public Health and Informatics, Bangabandhu Sheikh Mujib Medical University, Shahbag, Dhaka, 1000, Bangladesh; c BRAC Institute of Governance and Development (BIGD), BRAC University, Dhaka, 1212, Bangladesh

**Keywords:** COVID-19 pandemic, Celebration of death, Transition in tradition, Emotional reaction, Qualitative, Bangladesh

## Abstract

**Introduction:**

During the COVID-19 pandemic, restrictions significantly impacted religious activities related to death celebrations, mortuary practices, and funerary rituals in Bangladesh. Bereaved families faced a harsh reality, unable to perform the full rituals for their loved ones due to the pandemic outbreak. This study explores the adjustments and modifications made in cultural rituals and how they affected bereaved families and close relatives.

**Objective:**

This study aims to explore how the COVID-19 pandemic impacted the observance of death rituals in Bangladesh and the effects on bereaved families and close relatives.

**Methods:**

This study employed a qualitative approach and gathered data through 3 key informant interviews (KIIs) and 58 in-depth interviews (IDIs) conducted between December 2020 and January 2021. Purposive sampling was employed to recruit participants from three distinct groups: (1) individuals who had experienced the death of a relative during the pandemic, (2) caregivers who provided support to those experiencing loss, and (3) volunteers actively involved with funeral and burial services. This selection strategy ensured a diverse range of perspectives on the impact of the pandemic on death rituals.

**Results:**

The pandemic forced people to observe funerals or make significant sacrifices to traditional practices reluctantly. Disruptions to long-standing traditions and widespread emotional toll were as various segments of society struggled to find closure in saying goodbye to loved ones. Due to the severity of the pandemic, people became heavily reliant on priests, crematorium workers, and funeral volunteers for mortuary services. Both Hindu and Muslim religions discouraged family gatherings during post-burial rituals. The fear of contracting the virus further limited bereaved families’ ability to bid farewell to their deceased loved ones properly.

**Conclusion:**

This research highlights the profound impact of the pandemic on death rituals and the resulting emotional distress for bereaved families in Bangladesh. The findings are pivotal in guiding the development of concrete policies for future pandemic preparedness and response. Such policies might encompass guidelines for safe and culturally sensitive mortuary practices, psychosocial support and grief counseling initiatives for affected communities, and strategies to mitigate religious anxieties during public health emergencies.

## 1 Introduction

COVID-19 began to spread worldwide on the eve of 2020, prompting the adoption of mandatory preventative measures, particularly social distancing in most countries [1]. However, these restrictions adversely affected people's personal and social lives [2]. Every facet of human existence, including family and social life, has been altered by COVID-19 [3]. Studies have shown that society's cultures, including all faiths, belief systems, and ideologies, are intended to fend off the fear of death by using them as bulwarks and coping strategies [4,5].

During the COVID-19 pandemic, various countries implemented mandatory regulations and guidelines for burial and funeral rituals [6,7]. A UK-based longitudinal survey has shown that these measures encompass restrictions on the number of attendees and the inability to perform traditional rituals [8]. The imposed restrictions have caused distress and a sense of isolation among the bereaved, as they were unable to receive emotional support from their relatives and friends [9].

In Bangladesh, a low-middle-income country with one of the world's most densely populated areas [10], people were discouraged from participating in community gatherings and various cultural rituals, including funerals. They were encouraged to stay home, engage in social distancing, adhere to home quarantine guidelines, and adopt other protective measures due to the fear of COVID-19 [11]. However, how rituals for the deceased were conducted during the COVID-19 pandemic was also influenced by societal norms. In the absence of proper burials and funerals, volunteer groups (individuals or groups who willingly performed burials and funerals during COVID-19) stepped in to carry out the procedure.

Every culture has a unique way of observing funeral ceremonies or rituals involving the deceased's body. However, during the COVID-19 pandemic, adherence to these rituals was altered in most countries, resulting in the deceased not being buried with the dignity typically accorded [[12], [13], [14], [15]].

Moreover, family members, relatives, and neighbors of the deceased were discouraged from attending burials and cremations, further compounding their grief [16,17]. In light of these situations, this study aims to examine the reasons behind the alterations to burial and funerary customs and how they were performed during the COVID-19 pandemic in Bangladesh. The findings of this study may contribute to the development of community-based social policies and guidelines for future pandemic preparedness and response to ensure the safety of individuals in any pandemic situation.

## 2 Literature review

The impact of the Covid-19 pandemic on funeral customs has been substantial. Numerous countries have implemented stringent restrictions and guidelines for funerals and memorial services, including limitations on the number of attendees and restrictions on performing traditional rituals [8]. Consequently, this has resulted in distress and a sense of isolation for the bereaved, as they were unable to receive emotional support from relatives and friends [6]. Moreover, the disruption to memorialization practices has extended beyond the funeral itself, encompassing restrictions on cemetery visits and the inability to scatter ashes in chosen resting places [18]. The absence of physical contact with the deceased and the omission of traditional rituals have made the mourning process more difficult, complicating mourners’ ability to fully comprehend the reality and inevitability of death [19]. Although virtual networks have served as an alternative, they are often perceived as unreal and incapable of replacing the solace provided by physical presence [20]. Overall, the pandemic has profoundly altered funeral customs, leaving many to grapple with feelings of sorrow, remorse, and a sense that the lives of the deceased have not been adequately commemorated.

Distinguished ethnographic works by notable figures in the anthropology of death [[21], [22], [23]] have been reconsidered and reevaluation in recent decades [24]. These works have significantly contributed to comprehending “mortuary rituals, the emotions of grief and mourning, and the complexities of death and the afterworld” [24]. The contemporary anthropology of death no longer examines the concept of death as a distinct biological and sociocultural phenomenon. Instead, it investigates it in conjunction with life because the boundaries between them are perceived as permeable, and liminality is viewed more as a bridge connecting life and death rather than a delineated time frame [24].

Grief and loss theory is highly relevant to understanding the impact of COVID-19 deaths and funerals. The pandemic has presented unique circumstances that complicate the grieving process and disrupt traditional mourning rituals. The Dual Process Model (DPM) of grieving, as delineated by Stroebe and Schut [25], emphasizes the oscillation between loss-oriented (LO) coping and restoration-oriented (RO) coping. However, the pandemic has disturbed this natural oscillation, necessitating individuals to adapt their coping activities and behaviors, potentially resulting in long-term effects on bereavement outcomes [8]. Additionally, the concept of ambiguous loss, where the deceased is physically absent but psychologically present, has been applied to the COVID-19 pandemic. This particular type of loss further complicates grief and hampers the acceptance of the loss [20]. The erosion of coping resources, disruption of routines, and elimination of face-to-face mourning rituals due to the pandemic also contribute to the challenges encountered by bereaved individuals [26]. The absence of ceremonial practices and mourning rituals, coupled with the incapacity to engage in a formal bidding adieu, may lead to a state of disengagement from the experience of sorrow and the forfeiture of acknowledgment from society and culture. These factors consequently exert a profound influence on the process of grieving [27].

## 3 Methods and materials

### 3.1 Study design and procedure

The research adopted a qualitative content analysis technique that used purposive sampling to identify potential respondents. Participants were selected from several unions, the lowest administrative unit, in the Dhaka, Narayanganj, and Cumilla districts for data collection. The sample included relatives of individuals who had passed away due to COVID-19 (n = 14), caregivers of these deceased individuals (n = 22), and organizational volunteers involved in the burial processes of people who died from COVID-19 (n = 25).

We recruited three undergraduate students from the Department of Anthropology at Comilla University, all hailing from the districts targeted for data collection. Additionally, a field supervisor was recruited to monitor the team. The data collectors participated a three-day training session to prepare for the interviews, focusing on data collection techniques, preserving confidentiality, and taking note of non-verbal expressions. Prior to beginning the main interview phase, three pre-test interviews with diverse populations were conducted to refine the approach. Data were then collected from homes, clinics, and at the volunteer organization, encompassing each group involved in the study.

Data were obtained through key informant interviews (KIIs) and in-depth interviews (IDIs), with a total of 61 adults participating. Interview schedules were prepared for both KII and IDI, including questions about funeral practices before and during the COVID-19 pandemic (as outlined in Annexure A). The data collection adhered to the data saturation principle [[28], [29], [30], [31]]. Field notes were also taken during the interviews. The interviews spanned between 20 and 30 min each. On the interview day, data collectors recorded information on paper and subsequently made transcriptions. Before each interview, the data collectors explained the purpose of the study to the participants and obtained their written consent for the interview. The data were collected from December 2020 to January 2021.

### 3.2 Data analysis

**Fig. 1:**
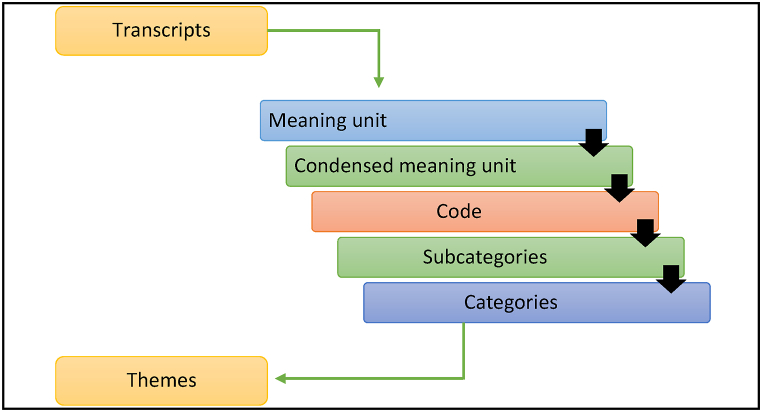
Qualitative data analysis techniques.

The data were analyzed using the qualitative content analysis method, focusing on manifest and latent content. This technique was applied throughout the analysis [32], as illustrated. (Fig. 1).

Two data coders, M.R.I and M.C., were assigned to code all the transcripts to ensure intercoder reliability. Before starting the coding process, they underwent training. The analysis began with a thorough reading of the written transcriptions to gain an overview of the data material. Subsequently, meaning unit phrases relevant to the aim of the study were selected from the texts then condensed them. These condensed meaning units were then coded and grouped into categories. The data were thematically categorized [33,34] prior to detailed analysis. The process of data analysis is depicted in Table 1.

**Table 1:** Qualitative data analysis process from transcript to generate theme.

Transcript	Meaning unit	Condense meaning unit	Extracted Code	Subcategory	Category	Theme
“Both my parents died in the hospital after staying in the ICU. They were admitted with COVID-19 symptoms, but they passed away before receiving the test results. I traveled to my village with the dead body of my parents for burial… villagers did not allow me to enter the village as they feared I or the dead bodies carrying the virus …” – (IDI-06)	Villagers seemed to think that the chance of being infected by COVID-19 was high among them, so they reacted to the deceased's family member.	Chance of being affected by COVID-19 from deceased body and who with them	Conception of being affected by covid-19	Affected by COVID-19	Community aversion	Society's reaction to dead bodies by COVID-19
“…. we (the volunteer team) went to the house of the deceased. It was about 10 p.m. since it was a village … The family did not keep the deceased's body in the house but in a bush outside the house and everyone stayed at home. No one was near the body, fearing being infected. We were very surprised to witness the incident … we bathed the dead body and performed the burial” – (KII-01)	The conception of family members being affected by covid-19, so that they were refused to attend the funeral	They were frightened of being affected by COVID-19	Misconception create fear among family members	Change of attitudes	Fear of contracting COVID-19	Impact of COVID-19 on the people's emotional reaction to the deceased

### 3.3 Data quality

To maintain data quality, researchers meticulously employed a range of rigorous techniques. Triangulation [35], the strategic use of multiple data collection methods like surveys, interviews, and observations, provided strong converging evidence and mitigated potential biases inherent in any single approach. Contextual familiarity [36], achieved through extensive immersion in the research setting, allowed for nuanced data interpretation and minimized misinterpretations based on limited understanding. Member checking [37], where participants actively verified the accuracy of findings, ensured authenticity and prevented researcher bias from distorting the data. Peer debriefing [38], seeking feedback from experienced colleagues, yielded valuable insights into potential methodological flaws and strengthened the overall research design. Finally, reflexivity [39], the critical self-examination of personal biases and assumptions, enabled researchers to maintain transparency and minimize the influence of their own beliefs on data analysis and interpretation. These combined efforts successfully safeguarded the quality and trustworthiness of the data, ultimately leading to robust and reliable research findings.

### 3.4 Strengths and limitations

The main strength of this qualitative study lies in its representative sample. The informants were recruited from diverse geographical settings in Bangladesh, with participants from Islam and Hinduism, providing us with a comprehensive view of COVID-19.

On the other hand, the main limitation is the availability of respondents. There was a challenge as the hospital authority refused to provide the list of individuals who had died from COVID-19. Consequently, we needed to find out the home address of the deceased. To overcome this obstacle, we utilized personal networking to identify potential participants. Thus, we found them through purposive sampling, but they were scared of being infected by COVID-19. Due to the pandemic, some respondents refused to participate in the interview. However, we ensured safety measures during the interviews. Another limitation was that participants had limited time to conduct the interviews, as they were busy with their professional duties as doctors, nurses, and funeral organizers. We conducted interviews and cross-checked data over the phone when further clarification was necessary. Another limitation is that we did not conduct any focus group discussions (FGD), which are essential to understanding the entire societal perspective on this study. However, we attempted to compensate for this by maximizing in-depth interviews to cover the matter comprehensively.

## 4 Results

The outcomes of the study delineated the perspectives of family members, caretakers, and volunteers, culminating in the identification of three overarching motifs: commemoration of the deceased, psychological responses to death, and societal responses to the deceased individual's remains following a COVID-19 fatality. Each of these motifs has manifested in a range of three to five classifications, with the analysis further enriched through a juxtaposition of conventional and altered funeral customs influenced by religious beliefs within the Bangladeshi setting.

### 4.1 Demographic characteristics of the study participants

The demographic characteristics of the study participants presented their place of residence, age, sex, religion, and education level. Table 2 shows that almost two-thirds of the participants were male because it was challenging to reach female participants due to the COVID-19 pandemic. Although we aimed for equal representation from every religion, we could only include Muslim and Hindu participants from the respective areas. However, we analyzed the findings by giving equal importance to both religious practices regarding mortuary and funerary customs. The data presented in the table indicates that individuals were recruited from various educational settings, ranging from secondary school to higher education, with merely three originating from primary education. The distribution of respondents and participants across districts was almost equal.

**Table 2:** Respondents’ Demographic characteristics.

	N	%
	61	
District		
Dhaka	17	27.9
Narayanganj	18	29.5
Cumilla	26	42.6
Sex		
Female	22	36.1
Male	39	63.9
Religion		
Islam	52	85.2
Hinduism	9	14.8
Age		
18−3	26	42.6
31−40	20	32.8
41−50	10	16.4
<50	5	8.2
Education		
Primary (1−5 years of schooling)	3	4.9
Secondary (6−10 years of schooling)	16	26.2
Higher Secondary (10−12 years of schooling)	11	18.0
Tertiary (more than 12 years of schooling)	31	50.8
Respondent category		
Relative of the deceased	14	23.0
Caregiver	22	36.1
Volunteer related to funerals and burials	25	41.0

^a^Key informant interview (KII) = 3, In-depth interview (IDI) = 58 (39 men, 22 women).

### 4.2 Impact of the COVID-19 pandemic on the celebration of death

Due to the rapid spread of COVID-19, individuals across various strata of Bangladeshi society encountered challenges in comprehending and mitigating its transmission, resulting in outbreaks. As COVID-19 infection was viewed as life-threatening, many individuals considered death as its unavoidable outcome. Yet, due to the restrictions imposed by the government to slow down the infection rates, relatives were not permitted to attend funerals. As a result, special teams of volunteers engaged in the burial process during the pandemic and performed funeral rites, bathing, and interment. Although beneficial to the family members, it did not ease the pain caused by the loss of a loved one. During our fieldwork conducted in three districts of Bangladesh from 2020 to 2021, it was noted that the traditional practices for burial and funerary rituals were not followed. We have compiled a list of compromised mortuaries and funeral rites in Table 3.

**Table 3:** Traditional and compromised funeral rites due to COVID-19 in Bangladesh based on fieldwork 2020–2021.

Characteristics	Religion	Traditional practices	Compromised practices during COVID-19
Bathing of the deceased	Islam	─ Purifying the body is mandatory in Islam ─ Family members and the nearest person participated ─ Professionals are hired to perform the bathing	─ Purifying is neglected in bathing ─ Most family members and the nearest people did not participate ─ Volunteer teams have performed the bath
	Hinduism	─ Family members, close friends, or funeral home personnel bath the body ─ The body is cleansed in a concoction of milk, yogurt, ghee (clarified butter), and honey in preparation for the *holy bath*	─ In most cases, family members solely depended on funeral home workers ─ Full Hindu funeral rites cannot always be followed due to the abundance of infected dead bodies
Performing *Janazah/cremation*	Islam	─ *Janazah* is performed in an open space ─ Family members, relatives, friends, and community members attend ─ A huge number of people gather for *Janazah*	─ *Janazah* is performed in an open place but attended by very few people. Sometimes no one was present ─ Family members, relatives, friends, and community members often choose not to attend ─ Only volunteers perform all the formalities
	Hinduism	─ Before cremation, Hindus usually have a brief wake ─ A man's forehead should be rubbed with ash or sandalwood, while a woman's forehead should be rubbed with turmeric ─ *Holy basil* should be placed in the coffin, and a floral garland should be draped around the deceased's neck	─ Organizing wakes for the deceased was practically impossible ─ Most bodies are carefully draped with white clothes while avoiding direct contact ─ In the absence of family members, crematorium workers recite hymns for the dead
Burial/cremation Proceedings	Islam	─ Usually, a burial place is selected for the community member ─ Family members are buried ─ Family graveyards are used	─ In many cases, deceased cannot be buried in their traditional burial places ─ Family members are not buried in many cases ─ Family graveyards are not used in many cases
	Hinduism	─ The body is taken to the cremation ground from home ─ The family builds a pyre and places the body on the pyre ─ A male family member sets the pyre on fire	─ Dead bodies are directly transported from hospitals since visiting home for the last time is discouraged ─ Priests and crematorium workers often take over this *holy duty* of cremation
Post-burial/Post-cremation Proceedings	Islam	─ Family members gather for a post-burial prayer ─ ‘Kulkhani’ (prayer for the dead after 40 days) is held	─ Family members do not gather for post-burial prayer in many cases ─ ‘Kulkhani’ (prayer for the dead after 40 days) is often not performed
	Hinduism	─ All family members will take a shower and put on clean clothing when they get home ─ A memorial ceremony called “sraddha” is arranged by the family after a few days to honor the deceased	─ Along with the cleansing ritual, earnest precautionary actions against COVID-19 are also performed ─ Family gathering is strongly discouraged ─ A proper memorial cannot be arranged due to safety reasons

The table reveals that during the observed procedures, we noted several changes. People have compromised their traditional practices due to COVID-19. The following subcategories provide detailed descriptions.

#### 4.2.1 Bathing of the deceased

In most cultures, substantial social connotation lies in giving a proper bath to a dead body. Within the Islamic faith, purifying the body is a mandatory custom because Muslims believe that a profane body causes pain to the soul. This ceremonial practice is also performed as a part of Hindu funerals. During the COVID-19 pandemic, professionals had to be hired to bathe, but relatives often participated as it is a person's last bath. Still, these practices were not always performed, as there is ample evidence of abandoned dead bodies that did not receive the last prayers or baths and were buried carelessly. In some cases, dead bodies were piled together and buried in a communal grave.

#### 4.2.2 Performing Janazah (Prayer)

As per Islamic customs the deceased has a definite right to a final bath. *Janazah* represents a distinctive social meaning, as neighbors visit the deceased's relatives to console them. These activities have been going on for decades, whereby *Janazah* would be performed in wide fields surrounding mosques and madrasas since large crowds generally gather to participate in prayer. Regrettably, this important custom had to be abandoned during the COVID-19 pandemic, due to which human feelings, compassion, and kindness were quickly replaced by self-absorption and indifference. In some instances, families did not even collect the dead body, leaving volunteers with the responsibility of washing the body and burying it while performing appropriate rituals.

#### 4.2.3 Burial proceedings

Burial is one of the crucial rites for every society, and its importance has unprecedented magnitude for different cultures. Within Bangladeshi Muslim societies, family graveyards are usually located in a place distant from their residence. Thus, relatives would gather at the grave to lay the dead body on the ground, grieve, and support each other. A volunteer said,

" … a person died of COVID-19 and his brothers did not come to the funeral and even the graveyard. His brother's sons was also forbidden from coming over." – (IDI-08)

These findings indicate that individuals experienced fear due to societal circumstances instead of necessary precautions. Consequently, volunteer groups expressed that they performed the burial proceedings and were engaged in burial and funerary processes for the sake of humanity.

#### 4.2.4 Post-burial proceedings

There are many rules about death in our society, including preparing for burial and praying for peace in the hereafter. In Islam, this also mandates that *“Kulkhani”* (prayer ceremony) be organized forty days following death. On this day, family members, friends, relatives, and neighbors gather to recite the *holy Quran* (Holly book of the Muslims). In practice, economic conditions determine the magnitude of the celebration, whereby affluent families would invite all villagers, while those with limited resources would struggle to organize even a small event. In such cases, they typically rely on the collaboration and sympathetic feelings of those close to them, and this sense of togetherness permeates the entire celebration. However, due to the constraints imposed by the COVID-19 pandemic, it became impossible to perform all customs as community members showed little interest even if the funeral was planned. Most people refused to participate because they were afraid of the pandemic. Still, as some informants articulated, given that visiting relatives without any obligatory reason was prohibited during the pandemic, organizing *Kulkhani* that would not be attended even by family members was pointless.

### 4.3 Impact of COVID-19 on people's emotional reaction to the deceased

The COVID-19 pandemic was an unprecedented, prompting most individuals to confront numerous critical situations, including the rules imposed on the traditional mourning for the deceased. As most healthcare systems worldwide were unprepared for the pandemic, some patients lost their lives due to carelessness and lack of attention. Bangladesh was not an exception, resulting in mistrust in the medical profession. On the other hand, due to fear of infection, some families refused to bury their loved ones, and this entire process had to be entrusted to the volunteers.

#### 4.3.1 Fear of contracting COVID-19

As noted previously, the COVID-19 pandemic induced fear of infection and death, which was further compounded by the constant updates in media regarding infection and fatality rates. As self-isolation and social distancing were portrayed as effective in preventing infection, those who had died of COVID-19 were often blamed by their relatives for being careless.

As indicated by many of our informants, the terror of death defeated human emotions. As a result, many people stopped performing their traditional rituals for fear of getting sick, as outlined by one volunteer:

“…. once I got a call to bury a man infected with the coronavirus. Upon receiving the news, we (the volunteer team) went to the house of the deceased. It was about 10 p.m. since it was a village, we felt like the night was getting darker. After reaching the destination, we saw a surprising scene. The family did not keep the deceased's body in the house but in a bush outside the house and everyone stayed at home. No one was near the body, fearing being infected. We were very surprised to witness the incident and without finding any other way, we bathed the dead body and performed the burial”. – (KII-01)

**Fig. 2:**
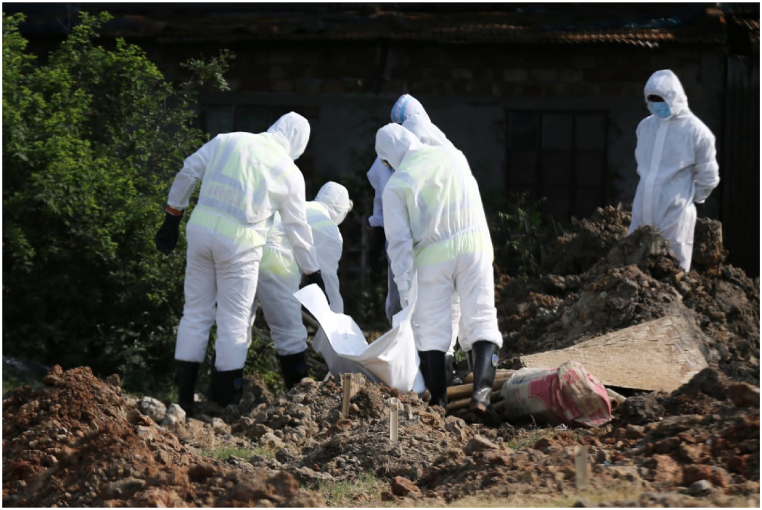
Burials during the COVID-19 pandemic, obtained from Dhaka Tribune.

These findings illustrated the fear and grief experienced by people during the pandemic, to the extent that they were unable to attend their loved ones' burials. Volunteers performed the burials, but they were not able to maintain proper dignity for the dead bodies. This witness testimony elucidates how the social reality was altered due to the pandemic. The pandemic has caused significant disruptions to people's lives and forced them to adapt to new ways of living, leading to changes in funeral customs and practices that have profound impacted people's ability to grieve and mourn their loved ones. Many pictures on social media have depicted the reality of COVID-19, as illustrated. (Fig. 2).

The figure expressed that due to the prevailing fear, patients who exhibited COVID-19 symptoms did not receive support from family members and often died as a result. In addition, those close to the deceased would negligently bid farewell to the ones with whom they had lived together, as aptly surmised by one of our informants:

“Initially, hospitals called us once a patient died from COVID-19. One late night we got a call from the hospital to arrange the burial of a body. When we reached the hospital, we could not find any relatives or family members of the deceased. After collecting the address, we took the body to their house. However, the family members did not even see the body and pointed out a distant spot for burial. We performed all the funerals and buried the deceased in a secluded place”. − (KII-01)

We found that fear was the most important factor that prevented people from attending burial rituals for their loved ones. They wanted to participate but were prevented from doing so.

#### 4.3.2 Irrational behavior in panic

The extensive COVID-19 pandemic induced widespread fear and anxiety, exacerbated by restricted movement that further complicated the procurement of essential goods, including food and hygiene products. Moreover, people began hoarding hand sanitizer and surgical masks, which led to shortages in healthcare facilities. People acted irrationally, as evident from the use of various “antidotes” that were not medically sound, causing further harm to public health. In some cases, relatives did not want to go near the body of a person who had died because of COVID-19 infection. One volunteer worker has highlighted that in this period, society's rules, regulations, and practices have often been neglected:

“I got a phone call from Saudi Arabia around 2 am (Bangladesh time). The person said his dead brother's body was lying in the hospital for a day but no one was willing to prepare a funeral and bury the body. We collected the body from the hospital and went to the deceased's home. None of the villagers came for the funeral. They only watched us from a distance. Even shopkeepers closed their shops once we passed in front of them in our ambulance. The village mosque also did not agree to give the khatiya (a small cot for carrying the dead body). Later, we arranged some alternatives and washed the body. The deceased's father also stood away from the body and was wearing PPE while we were in the village. He offered us some money after the burial. However, as a voluntary organization, we refused to take any money as we performed such humanitarian work without any return”. − (IDI-22)

Many were so scared and panicked at the beginning of COVID-19 that they stayed away from all social gatherings, including those with family members. The community acted insensitively due to widespread panic.

### 4.4 Society's reaction to dead bodies by COVID-19

We are all aware that death is the last step in a person's life, which should ideally be long and filled with various social relationships. These social bonds also imply responsibilities, commitments, and convey a sense of belonging to a community. That is why different rituals are observed when a person dies. Bereavement is a fundamental process in Bangladeshi culture, which includes people of many religious beliefs, and goes hand in hand with burial customs. Regarding this procedure, there are no rigid regulations. In Muslim communities, relatives come to comfort and feed grieving families. Similar rules apply to the Hindu faith, whereby relatives visit bearing food because cooking is prohibited for 24 h following a family member's passing. However, this practice was often abandoned due to the social distancing rule. As a result, the social and psychological processes associated with losing a loved one could not proceed as usual.

#### 4.4.1 Death and social life

During the COVID-19 pandemic, survival became the main objective, prompting many individuals to restrain their emotions. As a result, they have violated social rituals that have existed in society for hundreds of years. We encountered a limited cohort of individuals who were chosen not to follow the rules and rushed to the deceased's body, arranged the funeral, and conducted the burial proceedings. Indeed, in Bangladesh, volunteers worked tirelessly to ensure that these rituals were performed according to the proper customs. They could be contacted via social media or through SMS. As one volunteer explained:

“I, as a volunteer, attended 34 burials. However, in only six cases, I found family members of the deceased present at the time of burial. In other cases, only volunteers were the ones to take care of the dead body. Some family members only provided clothes for the dead body but did not accompany the body to the grave”. – (KII-02)

These findings illustrate how social life altered due to a pandemic death. People were terrified of being affected by COVID-19.

#### 4.4.2 Community aversion

As the COVID-19 pandemic has caused many social stigmas, some funeral ceremonies and burials were compromised. Several incidents occurred where community members declined to execute the last rituals of the affected dead bodies as they did not understand the virus transmission process. In some communities, in the face of growing panic, family members of the dead were denied the right to bury the body in their ancestral place. Intense terror of death turned into intense disgust and animosity, which was expressed explicitly in their attitudes toward COVID-infected patients, dead bodies, and even their families and relatives. A respondent stated:

“Both my parents died in the hospital after staying in the Intensive Care Unit. They were admitted with COVID-19 symptoms, but they passed away before receiving the test results. I traveled to my village with the dead body of my parents for burial. However, the villagers did not allow me to enter the village as they feared I or the dead bodies carrying the virus. I did not find any way to contact a volunteer organization. They later arranged the burial in an urban graveyard away from my village”. – (IDI-6)

The respondent further articulated that the local leader had stopped him from performing the burial in his village, and he was threatened with expulsion from the community and his family if he objected. These findings indicate that the inhumane reality forced many people to keep silent about their sickness.

#### 4.4.3 Sincerity of professionals

In the pre-COVID-19 era, people gathered in the same place to perform funerals for their loved ones, express grief, and console each other. It was relatively uncommon for someone to die alone with no one present to perform the last rites. However, the rituals underwent modifications in response to the COVID-19 pandemic, due to which many family members and relatives relied on volunteers. A volunteer explained who participated in many funerals during the COVID-19 pandemic, his team of six members once went to bury a Muslim's dead body. During the bathing of the dead person, family members brought hot water and clothes. Some of them took part in the *Janazah*. But when it was time to bury the body, none agreed to go to the graveyard. He stated:

“None of the family members agreed to catch the body to avoid getting infected. Even one from the Hindu religion of our team, went down the grave because they could not hold the body properly”. – (IDI-08)

These findings illustrate that the extreme transition of human emotions during COVID-19 is evident. Despite belonging to a different religion, a volunteer felt obliged to step in and ensure the dignity of the deceased. The same volunteer further stated:

“When fear was all around during COVID-19, some volunteer organizations were the only ones that came forward to bury the deceased. We took a lot of risks and did not even think about our own families but helped others. Once, we went to take care of a dead body and found that the body was left unattended in a bush. We made a funeral of the body and buried it in the graveyard with care. One team member was also infected with COVID-19 during the line of work”. – (IDI-08)

We observed that the volunteer team was incredibly empathetic in ensuring the dignity of the deceased during the final farewell. In their dedication to performing rituals steeped in tradition for hundreds of years, they denied health risks.

#### 4.4.4 Loss of communal empathy

Respondents of the study also described several incidents of family member abandonment at the time of need out of fear of infection for which there was no known cure. Connection and affection that had been growing for many years suddenly turned into some distant memories. Fear of death and yearning for survival made some of us self-centered. Moreover, health professionals and the government-mandated quarantine for people outside Bangladesh and those who have been diagnosed with COVID-19. However, some locals and villagers put a quarantine on anyone coming to their village from elsewhere. One respondent commented on this issue as follows:

“My office was closed, and I went to my village. I was forced to stay locked in my home for 14 days. My house had a limited supply, but I could not go to the market. I almost starved to death”. – (IDI-47)

The statement illustrates that communal empathy was lost during COVID-19. However, we found that the reality was not always so grim and harsh, as many volunteers tried their utmost to provide the required services without making any judgments about the deceased's religion or class because they felt that it was their responsibility to give a dignified funeral to each person.

## 5 Discussion

The COVID-19 pandemic cast a long shadow on mortuary and funeral practices in Bangladesh, a predominantly Muslim nation where customs and religious convictions hold immense significance. Compromising these practices, while essential for public health, triggered a complex interplay of grief, fear, and cultural anxieties among the family, relatives, and friends of deceased. The implications of these complex social circumstances are briefly discussed below.

**Disrupted rituals and heightened anxiety:** Bangladeshi Muslims adhere to specific rituals like ritual bathing (*ghusl*) and shrouding (*kafan*) before burial, emphasizing respect for the deceased and ensuring a smooth transition to the afterlife. So, death rituals are deeply ingrained in their faith and culture. Bathing the deceased, communal prayers, and elaborate burial ceremonies are integral to honoring the departed and providing solace to the bereaved. However, pandemic restrictions on gatherings and physical contact severed these vital connections. Limited family members at funerals, shortened prayer times, and the absence of traditional rituals left many feeling a sense of incompleteness and unfulfilled obligations towards the deceased. This, coupled with the fear of infection and the stigma associated with COVID-19 deaths, amplified the anxieties of grieving families.

**Navigating faith and public health:** Islamic teachings emphasize the importance of prompt burial (within 24 h) and respectful treatment of the deceased. However, concerns about viral transmission led to temporary modifications in traditional practices which is unacceptable in normal situations. The Bangladesh government issued guidelines for safe funeral procedures, including wearing masks, maintaining physical distance, and encouraging cremation in some cases (for Hindu communities). While these measures were crucial for public health, they also sparked debates within the community. Some viewed them as compromises of religious obligations, leading to confusion and resistance. Striking a balance between adhering to faith and ensuring public health became a delicate challenge for both religious authorities and the government.

**Finding solace in adaptation and resilience:** Despite the challenges, Bangladeshi communities displayed remarkable resilience in adapting to the pandemic's constraints. Families found innovative ways to uphold traditions, such as holding virtual prayers and sharing condolences online. Religious leaders issued guidance on safe practices while reinforcing the importance of respecting the deceased. This collective effort to adapt and find solace in modified rituals demonstrated the deep-rooted faith and cultural strength of the Bangladeshi people.

**Weaving grief through a fractured social fabric:** Traditional funerals in Bangladesh serve as an important social safety net, fostering community support and shared condolences. Large gatherings affirm communal bonds and provide solace for the bereaved. However, COVID-19 restrictions on gatherings severed these vital threads of support, leaving families to navigate their grief in isolation. The inability to perform customary rituals added to a layer of emotional anguish, as many felt they were failing in their religious and social obligations towards the deceased. This social isolation also exacerbated pre-existing inequalities, disproportionately impacting vulnerable communities who relied heavily on communal support during times of loss.

In many countries, especially at the start of the COVID-19 pandemic, mass funerals were not uncommon. Yet, this is not an unprecedented concept, as Lipton [40] observed the same practice in Freetown, Sierra Leone, during the 2014−15 Ebola epidemic.

However, in Bangladesh, certain customs that need to be performed during the funeral and burial of a deceased were abandoned during the COVID-19 pandemic, as initially people were frightened of touching the dead body and potentially getting infected. Consequently, volunteers from various religions assisted in the burial of COVID-19 victims, raising cultural expectations [41].

As pointed out in this study, during the pandemic, inhumane actions were not uncommon, as survival was the main priority. As a result, many COVID-19-infected dead bodies were left alone in a hospital corner with no one to look after them. These findings concur with the results reported by Radcliffe-Brown [42] a century ago based on a study on Andaman Islanders, revealing two kinds of weeping reciprocal and one-sided wailing. As the author explained, the objective of the custom was to uphold the presence of a social attachment between two or more people. Thus, the end of mourning was regarded as an association between mourners and the world of the dead. Likewise, in diverse societies of Bangladesh, people deem the funeral a last farewell to loved ones. Failing to do so disrupts the ritual and causes distress among the mourners for not being able to say the final goodbye.

During the COVID-19 pandemic, the rituals and rites that had existed in society for a long time and were centered on the dead individual were given little weight. In the pre-COVID-19 era, the inhabitants of this country had been engaged in arcane cultural practices, including rural and urban rituals concentrating on the dead individual. However, as revealed in this study, the pandemic disrupted emotional responses to the impacts of sickness, as the fear of infection took control of people's emotions.

Nonetheless, many volunteers were willing to risk their lives during the pandemic to bury the deceased, offer proper funerals, and perform all religious customs. In many cases, no family members were present. Still, this issue is not unique to Bangladesh, as in most countries, relatives were prohibited from attending funerals of their loved ones during some stages of the pandemic [43].

Fear-based stigmatization of Ebola in West Africa is merely similar to the turmoil wreaked by unforeseen consequences, as pointed out by O'Leary et al. [44] in their study on the fear of infection of an outbreak. In the present study, focus was given to the treatment of the diseased due to the fear of infection. However, in some cases, patients died alone as a result of inadequate care or neglect on the part of doctors and nurses. According to O'Leary et al. [44], Ahmed et al. [45], and Kamruzzaman [46], this is not surprising, as the initial fear caused by the unprecedented nature of the COVID-19 virus caused many to behave selfishly and disregard humanity. This fear was compounded by the emergence of new social regulations [11], as well as misinformation and rumors from numerous dubious sources.

As was shown here, in these circumstances, both Muslims and Hindus had to perform funerals in a compromised way. At the funerals of Muslims, their families and friends could not gather to commemorate the death of a loved one, as is customary. In Hinduism, the deceased's family was not present at the cremation. The special delegation team was in charge of these processes.

## 6 Conclusions and recommendations

The COVID-19 pandemic exposed the complex interplay between public health, religious traditions, and social structures in Bangladesh. While compromising traditional death rituals was necessary to curb the virus's spread, it also caused emotional distress and disrupted cultural practices. However, the pandemic also revealed the remarkable resilience and adaptability of communities, finding creative ways to honor the deceased under these challenging circumstances. Building on these insights, future research can explore several key areas:

**Policy Recommendations:** Informed by this study's findings, concrete policy recommendations can be developed for future pandemic preparedness and response. This could involve.

• Guidelines: Developing guidelines for safe and culturally-sensitive mortuary practices that balance public health needs with respect for traditions.
• Mental Health Support: Providing psychosocial and grief counseling initiatives to support communities affected by loss during pandemics.
• Religious Considerations: Implementing measures to address religious anxieties during public health emergencies, fostering collaboration between public health officials and religious leaders.

**Planning for Future Outbreaks:** Local stakeholders can analyze the gaps in healthcare and religious infrastructure exposed by the pandemic. This analysis can inform future planning and resource allocation to ensure a more robust and culturally sensitive response to future outbreaks.

**Community-Based Initiatives:** Community-based organizations and local NGOs can explore how community-led initiatives can support bereaved families and uphold religious practices while adhering to public health guidelines. This could involve.

• Training Religious Leaders: Providing training for religious leaders on safe burial practices conducted in accordance with public health protocols.
• Grief Counseling Programs: Developing culturally appropriate grief counseling programs to support those experiencing loss during pandemics.
• Public Awareness Campaigns: Promoting public awareness campaigns on safe mourning practices during pandemics, ensuring communities have the information they need to navigate these difficult situations.

## Ethics declarations

This study was funded, reviewed and approved by the research and extension division of Comilla University, Cumilla, Bangladesh, with the reference number: Ref. No.: COU/Reg./Research projects-485/2013/17,618. Date of issue: December 23, 2020. We also explained the purpose of the study to the participants and obtained written consent before the interview.

## Data availability statement

Data will be made available on request.

## CRediT authorship contribution statement

**Md Abdur Rahman:** Writing – review & editing, Visualization, Validation, Supervision, Resources, Project administration, Methodology, Investigation, Funding acquisition, Data curation, Conceptualization. **Md Redwanul Islam:** Writing – original draft, Visualization, Software, Resources, Project administration, Methodology, Investigation, Funding acquisition, Data curation, Conceptualization. **Monidipa Chowdhury:** Writing – original draft, Visualization, Methodology, Investigation, Funding acquisition, Data curation, Conceptualization. **Md Asaduzzaman:** Writing – review & editing, Visualization, Validation, Supervision, Project administration, Methodology, Investigation, Data curation, Conceptualization. **Proloy Barua:** Writing – review & editing, Validation, Supervision, Methodology. **M Atiqul Haque:** Writing – review & editing, Visualization, Validation, Supervision, Methodology.

## Declaration of competing interest

The authors declare that they have no known competing financial interests or personal relationships that could have appeared to influence the work reported in this paper.
