# Supplementary material for: Impact of COVID-19 on celebration of death, mortuary, and funerary customs in Bangladesh: A qualitative study
Source: Heliyon. 2024 Apr 27;10(9):e30369. doi: 10.1016/j.heliyon.2024.e30369 (PMC11076953; doi:10.1016/j.heliyon.2024.e30369)
Supplement: Multimedia component 1 [file mmc1.doc]

# **Annexure A: Supplementary materials**

**Interview guidelines**

Semi-structured guidelines for in-depth interviews and key informant interviews with bereaved close relatives, caregivers, and volunteers involved in mortuary and funerary customs during the COVID-19 pandemic

**Demographic Information:** Name, Sex, Age, Education, Address, Marital Status, Religion, Occupation

**Introductory Exchange:** Salam/Adab. Thank you for participating in this interview. This research is part of a project sponsored by the "University Grants Commission" at "Comilla University," focusing on changes in funeral rituals during the COVID-19 pandemic. The study aims to explore how societal practices surrounding death have evolved, analyzing both traditional and compromised post-mortem rituals. We delve into shifts in human behavior, societal norms, and the overall cultural landscape to provide insights into these cultural changes related to death.

Confidentiality is assured, and the information provided will be used solely for research purposes. Your identity will remain undisclosed, and interview data will be securely preserved. Feel free to stop the interview at any time. All responses will be treated as confidential and anonymous.

Do you have any questions before we begin the interview?

**Interview Guidelines for Bereaved Close Relatives**

1. How are you today amidst the COVID-19 pandemic situation?
2. Could you please explain how everyone behaved if someone in the family or neighborhood showed symptoms of COVID-19?
3. What was the behavior of relatives or neighbors after someone tested positive for COVID-19?
4. Explain the behavior of hospital doctors or staff with COVID-19 patients.
5. When someone you know has died of COVID-19, have you visited the place? (a) Yes (b) No (c) Comment:
6. If yes, could you please explain if there have been any changes in comparison to other times? (a) Yes (b) No (c) Comment:
7. If not, could you please explain why you did not go there?
8. During normal times, who is present during the bathing or washing of the deceased?
9. Who participated in burying or performing the last rites of the deceased during the COVID-19 period?
10. Have any specific prayers or rituals been performed for the deceased during COVID-19? If yes, what are they? (a) Yes (b) No (c) Comment:
11. Regarding the burial or last rites, what specific changes have been observed? Please provide details.
12. We have seen in various media that family members have left the deceased due to COVID-19. What is your opinion on why this has happened?
13. How has COVID-19 affected human emotions, even to the extent of compelling people to leave the deceased through various processes?
14. Can you elaborate on what kind of changes you observed in social interactions during the COVID-19 pandemic?
15. Could you explain what kinds of sentiments or feelings have you observed among people in society when proper post-death rituals are not followed?
16. If there are any specific observations regarding changes in post-death rituals during the pandemic, could you share some insights?

**Interview Guidelines for Caregivers of Bereaved**

1. How are you today amidst the COVID-19 pandemic situation?
2. Could you please explain what was the experience of the family members coming to the hospital after a COVID-19 patient was admitted?
3. When someone died of COVID-19, were the relatives present? If yes, how did you observe their behavior? If not, why were they not present there?
4. In the case of a COVID-19 patient's death, what observations have you made regarding the situation afterward?
5. How have you seen the family members express grief after the death of a COVID-19 patient?
6. Could you explain the activities of the burial team (volunteers) regarding the burial or cremation process of the deceased's body?
7. We have seen in various media that family members have left the deceased due to COVID-19. What is your opinion on why this has happened?
8. How has COVID-19 affected human emotions, even to the extent of compelling people to leave the deceased through various processes?
9. If you have witnessed any incidents during the pandemic involving emotions changing with the deceased, please provide a description.
10. If there is any more particular observation during the pandemic with a deceased person, please explain in detail.

**Interview Guidelines for Volunteers who Performed Mortuary and Funerary Customs during the COVID-19**

1. How are you today amidst the COVID-19 pandemic situation?
2. How did you work as part of a volunteer team or group during the COVID-19 burial process?
3. How did you receive news when someone died of COVID-19? Could you please explain the role of relatives when someone dies of COVID-19?
4. How was the behavior of the community people in case of someone's death from COVID-19?
5. Could you please explain how COVID-19 has brought changes in people's mutual behavior?
6. Could you explain what tasks you and your team completed for the deceased and their families?
7. During COVID-19, who were present from bereaved relatives at the funeral/last rites? How long did they stay there, and how was their behavior? Please describe it.
8. We have seen in various media that family members have left the deceased due to COVID-19. What do you think, and why have they left?
9. If you have witnessed any incident during COVID-19 involving emotions changing with the deceased, please describe it.
10. How have social relationships changed during COVID-19? Explain in detail.
11. If there is any more particular observation during the pandemic with a deceased person, please explain in detail.

…………………………………………………………………………..

Expressing Gratitude to Respondents

Ensuring Confidentiality

Any questions about the research
